# Supplementary material for: Study on the Role of Phytohormones in Resistance to Watermelon Fusarium Wilt
Source: Plants (Basel). 2022 Jan 7;11(2):156. doi: 10.3390/plants11020156 (PMC8781552; doi:10.3390/plants11020156)
Supplement: Supplementary file 1 [file plants-11-00156-s001.zip › Supplementary Material6 Table S4. Prediction of cis-acting regulatory element of 9 DEGs..pdf]

Supplement material 6

Table S4 Prediction of cis-acting regulatory element of 9 DEGs.

Gene ID	Gene name	Cis acting element	Locus function	Sequence	Number
Cla97C10G198890	*NPR*	ABRE	ABA	ACGTG	1
Cla97C01G009310		CGTCA-motif	MeJA	CGTCA	5
		TCA-element	SA	CCATCTTTTT	2
		TGA-box	IAA	AACGAC	4
		AuxRR-core		GGTCCAT	
Cla97C07G137510		TGA-box	IAA	AACGAC	2
		AuxRR-core		GGTCCAT	
		P-box	GA	CCTTTTG	1
		TCA-element	SA	CCATCTTTTT	2
Cla97C04G071000		ABRE	ABA	ACGTG	1
		TATC-box	GA	TATCCCA	1
		TCA-element	SA	CCATCTTTTT	1
Cla97C10G186260	*PYL*	CGTCA-motif	MeJA	CGTC, TGACG	2
Cla97C05G081110		ABRE	ABA	ACGTG	1
		CGTCA-motif	MeJA	CGTCA, TGACG	1
		TCA-element	SA	CCATCTTTTT	1
Cla97C09G172410		ABRE	ABA	GACACGTGGC, ACGTG, CACGTG	4
		CGTCA-motif	MeJA	CGTCA, TGACG	2
Cla97C09G174770		CGTCA-motif	MeJA	CGTCA, TGACG	2
		TATC-box	GA	TATCCCA	1
		TGA-element	IAA	AACGAC	1
Cla97C05G081210	*JAR1*	ABRE	ABA	CGCACGTGTC, ACGTG， AACCCGG	5
		AuxRR-core	IAA	GGTCCAT	1
		CGTCA-motif	MeJA	CGTCA，TGACG	4
		TGA-element	IAA	AACGAC	1
		P-box	GA	CCTTTTG	1
