# Supplementary material for: Study on the Role of Phytohormones in Resistance to Watermelon Fusarium Wilt
Source: Plants (Basel). 2022 Jan 7;11(2):156. doi: 10.3390/plants11020156 (PMC8781552; doi:10.3390/plants11020156)

Supplementary Material 1

Figure S1. Standard curve for detecting phytohormones.

A. Standard curve for detecting SA content; B. Standard curve for detecting JA content; C. Standard curve for detecting ABA content. SF7, Susceptible cultivar + FON, 7 days post inoculation (7 dpi); RF7, Resistant cultivar + FON, 7 days post inoculation (7 dpi). Data were expressed as mean ± SE (n=3).

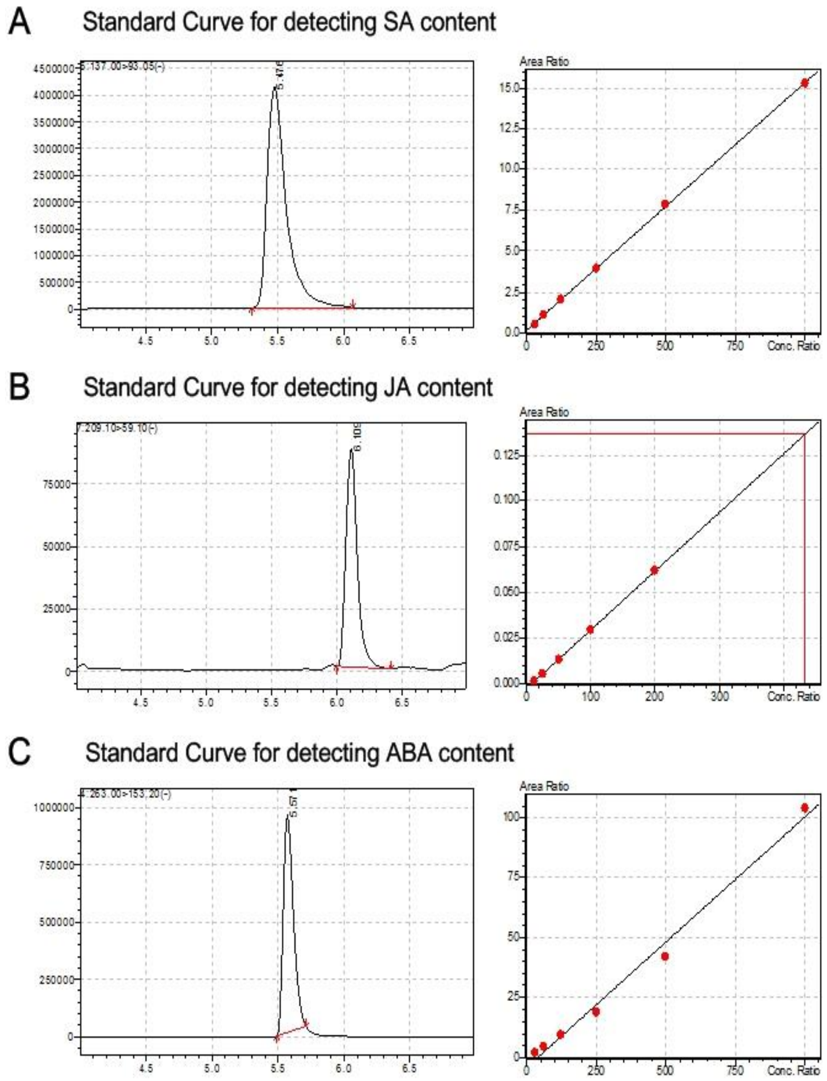

Supplement: Supplementary file 1 [file plants-11-00156-s001.zip › Supplementary Material1 Figure S1. Standard curve for detecting phytohormones..pdf]
