# Supplementary material for: Study on the Role of Phytohormones in Resistance to Watermelon Fusarium Wilt
Source: Plants (Basel). 2022 Jan 7;11(2):156. doi: 10.3390/plants11020156 (PMC8781552; doi:10.3390/plants11020156)
Supplement: Supplementary file 1 [file plants-11-00156-s001.zip › Supplementary Material2 Table S1. Quality control of sequencing data..pdf]

Supplementary Material 2

Table S1. Quality control of sequencing data.

Sample	Library	Raw_reads	Clean_reads	Clean_bases	Q20	Q30	GC_pct
SF7_1	FRAS202144495-1r	42753516	42282270	6.34G	98.31	95.1	43.89
SF7_2	FRAS202144496-1r	49744126	49206570	7.38G	98.39	95.25	43.54
SF7_3	FRAS202144497-1r	45134568	44562790	6.68G	98.34	95.22	43.79
RF7_1	FRAS202144501-1r	42417496	40933002	6.14G	98.3	95.13	43.35
RF7_2	FRAS202144502-1r	42528918	41896868	6.28G	98.12	94.82	43.39
RF7_3	FRAS202144503-1r	46896666	46195718	6.93G	98.04	94.58	43.19
