# Supplementary material for: Study on the Role of Phytohormones in Resistance to Watermelon Fusarium Wilt
Source: Plants (Basel). 2022 Jan 7;11(2):156. doi: 10.3390/plants11020156 (PMC8781552; doi:10.3390/plants11020156)

Supplementary Material 3

Figure S2. Function enrichment analysis of differential watermelon varieties. A. All genes KEGG enrich-ment significant analysis; B. Up regulated genes KEGG enrichment significant analysis; C. Up regulated genes GO enrichment significant analysis. Note: SF7, Susceptible cultivar +FON, 7 days post inoculation (7 dpi); RF7, Resistant culti-var+FON, 7 days post inoculation (7 dpi). Three independent replicates. Blue bands indicate low gene expression and red bands high gene expression.

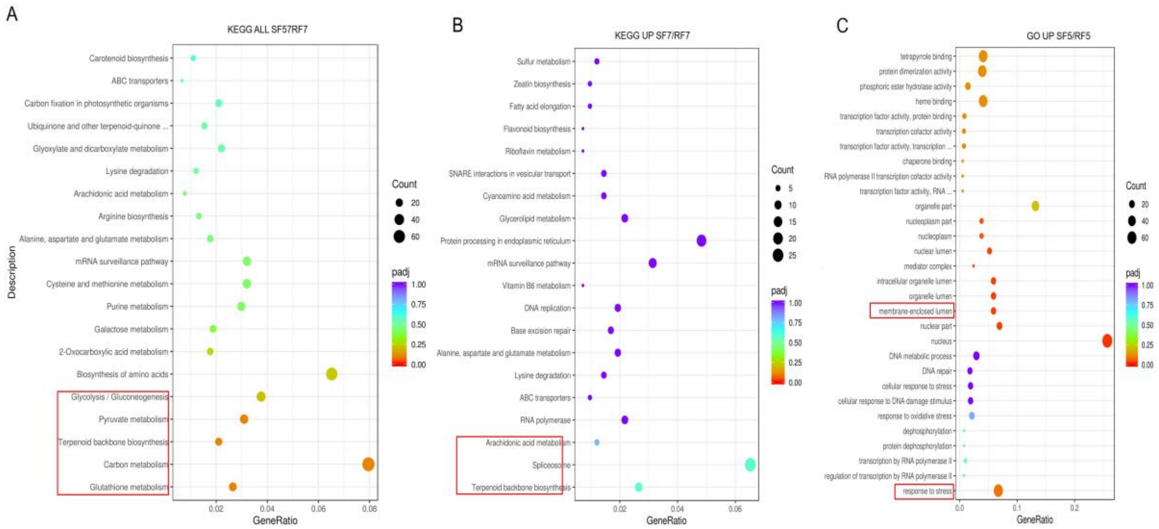

Supplement: Supplementary file 1 [file plants-11-00156-s001.zip › Supplementary Material3 Figure S2. Function enrichment analysis of differential watermelon varieties..pdf]
