# Supplementary material for: Study on the Role of Phytohormones in Resistance to Watermelon Fusarium Wilt
Source: Plants (Basel). 2022 Jan 7;11(2):156. doi: 10.3390/plants11020156 (PMC8781552; doi:10.3390/plants11020156)
Supplement: Supplementary file 1 [file plants-11-00156-s001.zip › Supplementary Material4 Table S2. Phytohormone DEGs.pdf]

Table S2 Phytohormone DEGs

gene_id	RF7_1	RF7_2	RF7_3	SF7_1	SF7_2	SF7_3
SA	Salicylic acid					
Cla97C06G125960	22.07107	13.50241	14.30004	10.46192	36.16392	36.49798
Cla97C02G047340	2441.305	1502.143	1827.341	2325.398	2896.066	2089.029
Cla97C03G065540	252.5911	291.4271	312.5581	365.2159	330.6416	121.0196
Cla97C04G075800	2572.506	2062.493	2847.752	3814.795	4206.085	3035.096
Cla97C04G075810	679.2984	401.6968	647.5877	1903.117	1530.693	883.6354
Cla97C04G075820	758.9995	547.9729	694.5736	1371.462	916.6447	419.7268
Cla97C04G075830	1840.482	2895.142	4673.05	4024.984	3096.813	2236.942
Cla97C04G075840	358.0418	926.0404	2020.392	1171.735	848.7451	312.1538
Cla97C04G075850	1101.101	2502.447	3080.638	3897.539	1586.046	2694.127
Cla97C04G075860	203.5443	30.38043	26.55723	499.3187	219.9357	198.818
Cla97C07G138590	102.9983	119.2713	132.7861	297.689	104.8016	90.28449
Cla97C07G138600	0	0	0	2.85325	0	0
Cla97C07G138620	41.6898	59.63565	117.4647	304.3466	144.6557	92.20543
Cla97C08G150340	9491.786	4301.643	5668.946	9147.518	7851.261	10762.1
Cla97C01G009310	984.6149	1297.357	1229.804	843.6108	745.4196	878.833
Cla97C10G198890	149.5928	351.0627	495.3944	308.151	333.5937	372.6636
Cla97C07G137510	2717.194	2804.001	2923.338	3050.124	2265.78	3438.494
Cla97C04G071000	0	0	0	8.559749	24.35529	5.76284
Cla97C05G099530	599.5974	678.4962	697.6379	1007.197	1079.013	739.5644
Cla97C06G126440	1830.673	2231.274	2203.228	2761.946	3096.075	1932.472
Cla97C10G197350	1276.443	1183.711	1538.276	2283.551	2374.272	1432.066
ABA	abscisic acid					
Cla97C06G123770	1630.807	2131.131	2241.021	367.1181	484.1537	480.2366
Cla97C05G102850	68.66555	38.25683	92.95029	594.427	526.96	688.6593
Cla97C09G174770	338.423	270.0482	338.0939	64.67366	69.37569	60.50982
Cla97C08G146170	261.1743	194.6598	704.7879	25.67925	30.25961	13.44663
Cla97C09G181450	164.3068	130.5233	112.3575	602.0357	693.0188	484.0785
Cla97C10G186260	4443.642	5522.487	5823.182	1641.57	1050.968	1627.042
Cla97C02G032900	94.41513	31.50563	106.2289	1296.326	418.4682	942.2243
Cla97C10G197930	171.6639	31.50563	101.1217	1030.974	315.8808	780.8648
Cla97C01G023840	2236.535	2110.877	2342.143	1171.735	1098.202	1312.006
Cla97C11G221400	506.4084	723.5042	741.5595	196.8742	322.5231	178.648
Cla97C09G182000	102.9983	95.64208	132.7861	272.9609	322.5231	236.2764
Cla97C08G145920	4.904682	12.37721	8.171454	35.19008	239.8627	28.8142
Cla97C08G158420	98.09364	118.1461	181.8149	47.55416	35.42588	22.09088
Cla97C01G010380	717.3097	909.1624	817.1454	461.2754	405.9216	450.462
Cla97C03G052230	702.5957	176.6566	336.0511	2772.408	1030.303	1142.963
Cla97C07G142130	0	0	4.085727	41.84766	38.37804	24.9723
Cla97C10G188860	1560.915	1135.328	1194.054	714.2635	783.0596	738.6039
Cla97C09G172410	977.2579	963.1721	1384.04	648.6387	608.8824	603.1772
Cla97C03G063210	1199.195	1166.833	1162.389	712.3613	805.9388	546.5093
Cla97C01G020790	3206.436	4160.993	3935.577	1844.15	1310.758	2700.851
Cla97C08G159130	279.5669	261.0466	315.6224	677.1712	364.5914	664.6475
Cla97C05G081110	1216.361	792.1415	1119.489	1772.819	2046.583	1567.492
Cla97C05G106700	1591.569	1520.147	2331.929	880.703	771.989	1282.232
Cla97C07G134120	1392.93	1295.106	1612.841	855.9749	753.538	1046.916
Cla97C11G223740	129.9741	108.0193	110.3146	215.8959	290.0494	179.6085
Cla97C07G139810	187.6041	123.7721	120.5289	328.1237	304.8102	220.9088
Cla97C11G223740	129.9741	108.0193	110.3146	215.8959	290.0494	179.6085
Cla97C07G139810	187.6041	123.7721	120.5289	328.1237	304.8102	220.9088

Cla97C08G151380	470.8495	382.5683	418.787	622.0084	669.4016	685.7779
Cla97C08G159620	7265.06	3476.871	3901.869	3036.809	2447.338	2543.333
Cla97C06G114490	0	0	0	14.26625	11.80863	2.88142
Cla97C07G137840	3332.731	2087.248	2099.042	3426.753	4807.587	4267.383
Cla97C05G087760	1092.518	417.4496	479.0515	365.2159	182.2957	342.889
Cla97C08G147150	565.2646	586.2297	651.6735	875.9476	1363.158	791.43
Cla97C01G006610	1329.169	1344.615	1411.619	911.1377	617.0008	1039.232
Cla97C04G068530	171.6639	172.1558	620.0091	1071.871	428.8008	947.0266
Cla97C06G123840	958.8653	856.278	687.4236	1397.141	1030.303	1457.998
JA	Jasmonic acid					
Cla97C07G130430	339.6492	324.0579	224.715	98.91265	73.80392	33.61656
Cla97C02G047730	149.5928	135.0241	221.6507	463.1775	389.6847	450.462
Cla97C10G204180	586.1095	446.7048	554.6374	1186.001	1374.229	899.003
Cla97C10G192210	7126.503	7245.169	7130.615	3631.236	3018.58	4780.275
Cla97C08G161000	77.24874	51.75925	92.95029	201.6296	287.8353	139.2686
Cla97C09G174730	2688.992	3179.818	3593.397	1877.438	1813.362	2085.187
Cla97C07G137410	56.40384	45.00804	51.07159	155.9776	82.66039	183.4504
Cla97C05G081210	973.5794	1160.082	1757.884	694.2907	723.2784	610.861
Cla97C08G158240	1743.614	2967.155	1611.819	1281.109	1049.492	663.687
Cla97C10G202820	689.1078	805.6439	893.7528	1257.332	1758.747	1166.975
Cla97C03G051780	486.7897	459.082	436.1514	743.7471	936.5718	640.6357
Cla97C01G011780	40.46363	79.88927	185.9006	30.43466	27.30745	27.85372
Cla97C02G045460	19.61873	45.00804	14.30004	79.89099	90.77882	77.79833
Cla97C05G108680	678.0723	703.2506	819.1883	1052.849	1075.323	1225.564
Cla97C06G114200	296.7333	473.7096	569.9589	854.0727	742.4675	787.5881
Cla97C10G186220	9732.115	9696.982	9380.829	6934.348	6844.576	4433.545
Cla97C06G115680	489.242	632.363	517.8659	944.4256	1190.457	674.2522
Cla97C05G100290	34.33277	24.75442	73.54309	161.6841	60.51922	154.6362
Cla97C05G100270	668.2629	428.7016	509.6945	984.3711	915.9067	712.6712
Cla97C02G035580	176.5685	277.9246	315.6224	421.3299	400.0173	489.8414
Cla97C05G100240	74.7964	79.88927	126.6575	58.96716	38.37804	24.01183
Cla97C04G078620	5396.376	5135.417	4514.728	1998.226	2039.94	2101.515
Cla97C05G105650	3839.14	3390.231	2356.443	1052.849	831.7702	1009.457
Cla97C05G100320	44.14214	73.13806	40.85727	0	0	0
IAA	Auxin					
Cla97C05G085700	1598.926	1849.83	3064.295	797.0077	593.3835	642.5566
Cla97C04G077140	371.5297	328.5587	396.3155	119.8365	62.73333	125.822
Cla97C10G197930	171.6639	31.50563	101.1217	1030.974	315.8808	780.8648
Cla97C02G050560	277.1145	355.5635	364.6511	654.3452	1111.487	706.9083
Cla97C11G221050	367.8511	234.0418	273.7437	598.2313	876.7906	627.189
Cla97C02G034980	239.1032	407.3228	350.3511	165.4885	80.44627	67.23313
Cla97C09G173780	29.42809	48.38364	16.34291	103.6681	191.8902	95.08685
Cla97C02G042430	586.1095	322.9327	457.6014	793.2034	1157.984	828.8884
Cla97C08G155010	6479.085	4970.013	5237.902	8330.538	9921.461	8665.39
Cla97C01G001810	2021.955	2365.173	2030.606	1018.61	1642.137	930.6986
Cla97C11G217540	2706.158	3715.414	3207.296	6003.237	5097.637	4322.13
Cla97C06G116140	2709.837	2299.911	1931.527	3369.688	4093.165	3410.641
Cla97C05G107110	0	0	0	7.608666	2.952157	18.24899
Cla97C09G172320	2034.217	2986.283	2294.136	1266.843	1867.977	1373.477
Cla97C06G119700	24.52341	37.13163	39.83584	180.7058	196.3184	129.6639
ET	ethylene					

Cla97C03G065080	197.4134	164.2793	104.186	450.8134	1013.328	530.1812
Cla97C06G114420	13312.53	15878.84	20273.38	5521.989	4842.275	8277.359
Cla97C05G099120	614.3114	479.3356	539.316	1179.343	1082.704	1080.532
Cla97C10G193050	245.2341	452.3308	593.4519	138.8581	126.9427	85.48212
Cla97C10G197740	391.1484	410.6984	405.5084	194.9721	117.3482	155.5967
Cla97C04G078020	1750.971	1711.431	1498.44	754.209	957.9749	753.011
Cla97C05G104630	102.9983	130.5233	139.9362	301.4934	262.0039	403.3988
Cla97C08G149800	57.63001	4.500804	9.192886	384.2376	132.8471	158.4781
Cla97C08G159750	318.8043	228.4158	343.2011	104.6192	132.8471	128.7034
Cla97C09G170370	9.809364	29.25523	8.171454	77.98882	78.23216	122.9406
Cla97C07G141200	1136.66	1102.697	973.4245	1881.243	1768.342	1902.698
Cla97C10G193550	685.4293	630.1126	610.8162	1157.468	1079.013	1612.635
Cla97C02G032880	317.5782	344.3115	298.2581	480.297	886.3851	784.7067
Cla97C11G223860	13855.73	11529.93	9352.229	5485.848	6966.352	3818.842
Cla97C01G023310	617.9899	479.3356	316.6438	179.7547	119.5624	230.5136
Cla97C01G004570	26.97575	60.76085	107.2503	297.689	126.2047	377.466
Cla97C10G192160	1232.301	1682.175	1273.725	763.7198	853.9114	762.6158
Cla97C02G027280	125.0694	25.87962	59.24304	10.46192	16.9749	4.802366
Cla97C05G104890	106.6768	130.5233	348.3082	633.4214	505.5569	571.4816
Cla97C01G021180	87.0581	337.5603	139.9362	68.47799	39.11608	47.06319
Cla97C08G145370	0	2.250402	11.23575	45.65199	73.80392	26.89325
Cla97C03G060820	88.28427	126.0225	231.865	496.4654	410.3498	285.2606
Cla97C10G202480	521.1224	255.4206	323.7939	910.1866	993.4008	592.612
Cla97C01G003210	4.904682	15.75281	20.42864	33.28791	73.80392	106.6125
Cla97C02G043890	99.31981	109.1445	191.0077	375.6779	238.3867	327.5214
Cla97C06G121480	1477.535	1345.74	1430.004	917.7953	913.6926	632.9519
Cla97C01G001300	73.57023	29.25523	23.49293	8.559749	8.118431	4.802366
Cla97C02G043940	1033.662	1004.804	862.0884	578.2586	684.9004	577.2444
Cla97C05G108770	8.583193	10.12681	8.171454	0	0	0
Cla97C06G111500	1449.333	1534.774	1617.948	2244.556	2035.512	2504.914
Cla97C11G214720	2.452341	10.12681	14.30004	53.26066	149.0839	4.802366
Cla97C06G111000	1443.203	678.4962	1267.597	2501.349	1705.609	2168.749
Cla97C04G071960	28.20192	67.51206	82.73597	21.87491	1.476078	0
Cla97C11G221460	193.7349	182.2826	279.8723	430.8407	1064.991	195.9365
Cla97C05G099100	825.2127	461.3324	537.2731	1083.284	1116.653	877.8726
Cla97C01G023060	8128.284	6591.427	5920.219	9427.137	15419.12	9192.69
Cla97C05G089740	68.66555	51.75925	222.6721	140.7603	504.0808	332.3237
Cla97C09G169010	127.5217	113.6453	168.5362	293.8847	225.102	203.6203
Cla97C07G129280	36.78511	51.75925	70.47879	174.9993	67.89961	125.822
Cla97C04G072260	74.7964	94.51688	28.60009	144.5646	247.2431	97.0078
Cla97C06G128180	94.41513	93.39168	162.4077	204.4829	146.8698	401.4778
Cla97C03G063660	709.9527	917.0388	874.3456	609.6443	538.0306	582.0468
Cla97C05G085160	79.70108	49.50884	63.32877	1108.012	822.9137	777.0229
Cla97C05G087870	117.7124	111.3949	124.6147	747.5514	814.7953	865.3864
Cla97C05G080020	22.07107	19.12842	36.77154	171.195	369.7576	339.0471
Cla97C08G146560	49.04682	19.12842	29.62152	1038.583	150.56	705.9478
Cla97C05G105650	3839.14	3390.231	2356.443	1052.849	831.7702	1009.457
Cla97C10G194030	2219.369	2742.115	2220.593	1093.746	1100.416	860.584
GA	gibberellin					
Cla97C05G099610	67.43938	95.64208	66.39306	15.21733	14.02275	5.76284
Cla97C03G058120	30.65426	11.25201	12.25718	172.1461	41.3302	62.43076
Cla97C01G024580	45.36831	56.26005	147.0862	15.21733	25.09333	21.13041
Cla97C05G099600	220.7107	247.5442	332.9868	142.6625	125.4667	48.02366

Cla97C08G145860	652.3227	262.1718	243.1008	161.6841	222.1498	123.9011
Cla97C06G112650	193.7349	70.88766	134.829	505.9763	157.2024	315.9957
Cla97C07G137160	9.809364	10.12681	8.171454	125.543	91.51686	104.6916
Cla97C07G143880	12.2617	38.25683	39.83584	351.9008	208.1271	249.723
CTK	Cytokinin					
Cla97C10G202900	305.3164	288.0515	224.715	972.007	808.1529	1390.765
Cla97C08G152350	1622.224	358.9391	1232.868	6618.588	3542.588	4631.402
Cla97C11G208110	215.806	286.9263	397.337	116.9832	109.9678	125.822
Cla97C02G040700	29.42809	29.25523	3.064295	0	0	0
Cla97C05G099290	1607.509	1601.161	1651.655	585.8673	1037.683	722.2759
Cla97C09G166350	104.2245	381.4431	128.7004	19.02166	46.49647	56.66792
Cla97C05G090160	125.0694	77.63887	104.186	38.04333	32.47373	11.52568
Cla97C10G198530	521.1224	423.0756	307.451	176.9015	231.0063	198.818
Cla97C05G090160	125.0694	77.63887	104.186	38.04333	32.47373	11.52568
Cla97C10G198530	521.1224	423.0756	307.451	176.9015	231.0063	198.818
Cla97C11G207380	2427.818	4984.64	3273.689	1700.537	2023.704	1846.99
Cla97C11G212390	11.03553	38.25683	26.55723	4.755416	5.166275	4.802366
Cla97C06G115310	212.1275	133.8989	143.0004	98.91265	20.6651	60.50982
Cla97C02G047290	44.14214	93.39168	71.50022	193.0699	208.8651	84.52165
Cla97C10G201490	58.85618	72.01286	85.80027	43.74983	23.61725	12.48615
