# Supplementary material for: Study on the Role of Phytohormones in Resistance to Watermelon Fusarium Wilt
Source: Plants (Basel). 2022 Jan 7;11(2):156. doi: 10.3390/plants11020156 (PMC8781552; doi:10.3390/plants11020156)
Supplement: Supplementary file 1 [file plants-11-00156-s001.zip › Supplementary Material5 Table S3. Specific primer sequences of 10 candidate genes used for qRT-PCR.pdf]

Supplementary Material 5

Table S3. Specific primer sequences of 10 candidate genes used for qRT-PCR

**Gene ID**	**Gene name**	**Forward primer (5'-3')**	**Reverse primer (5'-3')**
Cla97C04G073730	*Clathin light chain*	CGTTCAACCAGGGAGACGAA	TGGCGCATTCTCGAGAGATC
Cla97C09G174770	*Abscisic acid receptor PYL*	AAGGGCGAACCGTCAGAAAT	AGACGACGGTCCAGATCTGA
Cla97C05G089520	*PP2C*	AGGTCACGTGAGATGGAGGA	CCTTCAACCCGAGCTCCATT
Cla97C05G081210	*JAR1*	TGGGCTGATACCAGAGCTCT	TCCCCGCTTATCTCCCAGAA
Cla97C01G009310	*NPR1*	GCTGGCAATGGCTGATGATG	CTGGGATTGGCACCATCCTT
Cla97C10G198890	*NPR5*	TCTTGGCGGCTAGGAGTTTG	GTCGTGATGGTGGTGGTGAT
Cla97C07G137510	*NPR1-putative*	TCATTGTGGCCCTTCTGGAC	AACCGAGCCTTCTTCTTCCG
Cla97C04G071000	*NPR5-like protein*	CGTCGATCTCGCTCTCGAAA	GCGATGTGCAAGGGTGTTTT
Cla97C05G099530	*BAH domain-containing protein, putative*	TTGCTCGGCAAGTAGCTCAA	ACTTTACGTGGGGAAGCAGG
Cla97C10G197350	*BAH*	TCCCTTGATGAAATGGGGGC	TGAATAAGGGGTCGGGGCTA
Cla97C04G074880	*CIACT*	CCCTTTATGCCAGTGGTCGT	TGGCGCAACCACCTTAATCT

Note: The watermelon *CIACT* gene was used as reference gene.
